# Viral involvement in the pathogenesis and clinical features of ophthalmic pterygium (Review)

**DOI:** 10.3892/ijmm.2013.1438

**Published:** 2013-07-12

**Authors:** AIKATERINI K. CHALKIA, DEMETRIOS A. SPANDIDOS, EFSTATHIOS T. DETORAKIS

**Affiliations:** 1 Department of Ophthalmology, University Hospital of Heraklion, 71110 Heraklion, Crete, Greece; 2 Department of Virology, University Hospital of Heraklion, 71110 Heraklion, Crete, Greece

**Keywords:** pterygium, cornea, HPV, virus, pathogenesis

## Abstract

Pterygium is a potentially vision-threatening fibrovascular lesion originating from the conjunctiva that often extends on the corneal surface. Although it has been extensively studied, its pathogenesis has yet to be fully elucidated. Recent evidence on molecular genetic abnormalities in pterygium suggested neoplastic changes of limbal stem cells potentially associated with exposure to ultraviolet (UV) light. Human papillomavirus (HPV) is an oncogenic virus, associated with squamo-proliferative lesions of the anogenital region, skin and oropharynx. Several studies have shown HPV involvement in the pathogenesis of conjunctival neoplastic lesions, including papilloma and squamous cell carcinoma. The involvement of HPV as a co-factor in the pathogenesis of pterygium, although suggested by several studies using PCR and immunohistochemical techniques, remains controversial. Moreover, a marked variation in the prevalence of HPV in ophthalmic pterygium has been reported by different studies. Ethnic susceptibility and methodological differences in the detection of HPV may account for this variation. Surgical excision, often using sophisticated techniques, is the standard current method of therapy for pterygium. However, recurrences are frequent and recurrent lesions tend to be more aggressive. If indeed HPV is involved in pterygium pathogenesis or recurrence, anti-viral medications or vaccination may be new options in pterygium therapy.

## 1. Introduction

**Figure 1:**
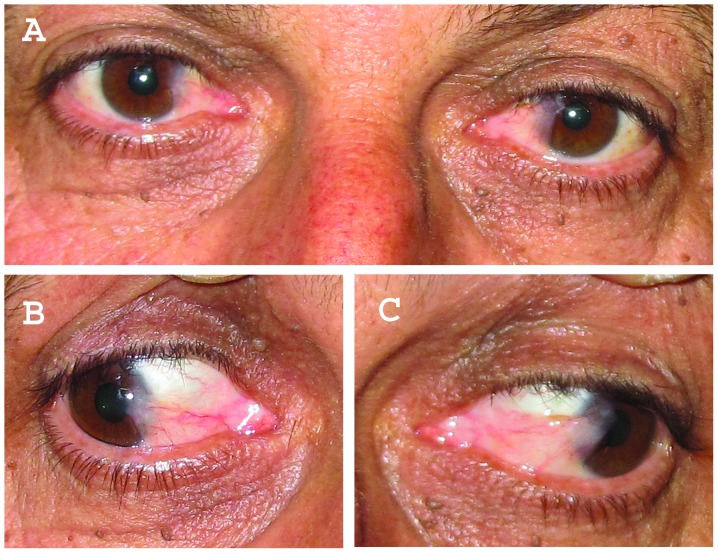
(A) Nasal bilateral pterygium. (B and C) The extent of advancement of the lesions on the corneal surface of the right and left eye is shown on abduction.

**Figure 2:**
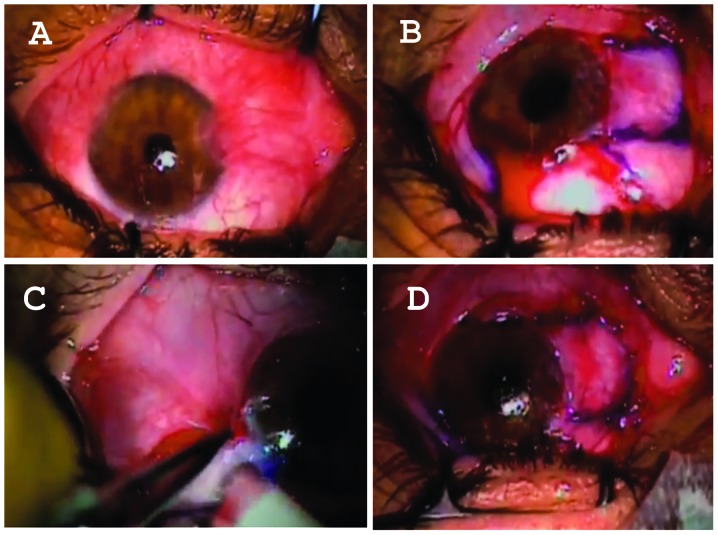
(A) Intraoperative views of nasal and temporal pterygium excision. (B) The nasal pterygium is excised and the defect covered with a conjunctival autograft harvested from the superior bulbar conjunctival area. (C) The temporal pterygium is excised and the defect covered with a locally raised conjunctival flap. (D) Final view at the conclusion of the procedure.

Pterygium is a wing-shaped fibrovascular lesion of the ocular surface of unknown origin and pathogenesis. It grows at the inter-palpebral conjunctival area, more often nasally, and invades adjacent corneal epithelium (Fig. 1). Pterygium growth can cause irregular astigmatism, corneal scarring, restriction of ocular motility, or chronic ocular surface inflammation (1). Treatment thus far is exclusively surgical, however, pterygium often tends to recur aggressively. This has lead to the use of sophisticated surgical techniques in an effort to reduce recurrence rates (Fig. 2) (2).

Although pterygium pathogenesis remains an enigma, epidemiologic studies suggest that it is a sun-related eye disease (ophthalmoheliosis). In fact, the prevalence of pterygium seems to be associated with geographical latitude, with higher prevalences in areas located within 40º above and below the equator, suggesting that prolonged exposure to ultraviolet (UV) radiation may promote its development. In addition, repeated micro-trauma, mediated by exposure to dust, chronic conjunctival inflammation, genetic predisposition and ocular dryness have all been reported to be involved in pterygium, indicating a multifactorial pathogenesis (1,3). Evidence suggests that genetically altered limbal epithelial stem cells may play a critical role through a process of Bowman's layer dissolution, matrix remodeling, cell proliferation and angiogenesis, possibly with the involvement of cytokines, matrix metalloproteinases and growth factors (4–10). Notably, among the various theories proposed for pterygium pathogenesis thus far, the possibility of an infectious (probably viral) mechanism in at least a subgroup of pterygia has also recently gained ground. This possibility is important because, should it prove valid, it could justify non-invasive or minimally invasive treatment options through anti-viral medications.

## 2. Theories of infectious pathogenetic co-factors in pterygium

**Figure 3:**
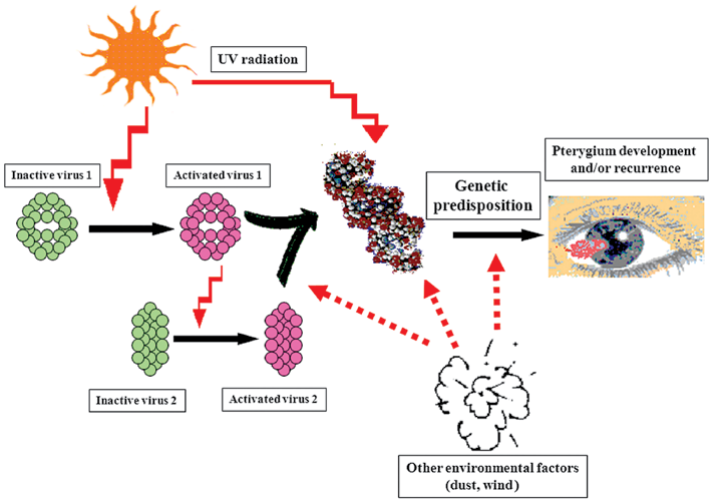
A proposed multi-step model for pterygium pathogenesis, in which genetic predisposition, viral involvement and environmental factors co-participate (adapted from ref. 15).

Pterygium has been previously considered a degenerative condition. This hypothesis, however, has been challenged in recent years by the detection of important molecular genetic alterations in pterygium, including loss of heterozygosity (LOH) of microsatellite DNA or the overexpression of mutated versions of p53 with compromised function, which could promote tumor growth (11–14). Accordingly, a multi-step pathogenetic process, with the participation of genetic inheritance, UV radiation and, importantly, oncogenic viral infection has been proposed for the pathogenesis of pterygium (Fig. 3). According to this ‘two-hit’ hypothesis, inherited genetic alterations or exposure to environmental factors such as UVR could predispose individuals to this benign neoplastic disease (‘first hit’). Oncogenic viruses, or additional UVR exposure, adding further damage to an already susceptible genetic material, may be the stimulus for the outset of development or recurrence of pterygium (‘second hit’) (15). This theoretical model has been recently supported by the detection of HPV strains considered high-risk for cancer development, including 16 and 18, encoding E6 and E7 proteins which interfere with p53 function (16,17).

## 3. Virus detection in pterygium

Many studies have been conducted to investigate the involvement of a variety of oncogenic viruses, including HPV, CMV, HSV or EBV, in the development and recurrence of pterygium (Table I). HPV presence in pterygium has been reported by several studies, with prevalence ranging from very low to almost 100% of cases (16,18–27). The prevalence of various herpes-viruses in pterygium also differs considerably among reports. Studies from Greece detected HSV in 22–45% and CMV in 40% of examined pterygia (18,28). On the other hand, in a study conducted in Taiwan Chen *et al* investigated the role of HSV in pterygium, where a prevalence of only 5% was reported (29). Another study from Turkey detected EBV-DNA in 10% of the pterygia examined (25). Such a disparity in the prevalence of oncogenic virus detection in pterygium may partly be explained by ethnic or geographical factors or by laboratory techniques. However, it may also reflect the heterogeneous nature of pterygium pathogenesis and the possibility that oncogenic viruses affect only a sub-group of ophthalmic pterygia.

## 4. Current status on HPV detection in pterygium

Taking into account that HPV is by far the most commonly reported oncogenic virus associated with ophthalmic pterygium, several studies have focused on HPV detection and typing to assess the potential role HPV plays in the pathogenetic process leading to pterygium development. Of note, certain studies (such as those of Sjö *et al* from Denmark, Takamura *et al* from Japan or Hsiao *et al* from Taiwan) have failed to detect HPV or report very low prevalences of HPV in examined pterygia (21–23,25,27). Moreover, Dushku *et al* detected p53 overexpression in the limbal epithelium of the pterygia studied with all the samples being negative for HPV DNA, suggesting that factors other than HPV infection were responsible for the p53 overexpression (12). To investigate the role of HPV and the variance in its prevalence in pterygium in the different studies, Piras *et al*(20) selected patients from two distant geographically populations, Italy and Ecuador. HPV was detected in all the pterygia of Italian patients, but in only 21% of the pterygia from Ecuador. In that study, geographic and ethnic factors were proposed as a possible explanation for differences in HPV prevalence in pterygium, supporting its multi-factorial pathogenesis (20).

## 5. HPV detection in ocular surface lesions

Over the past three decades HPV DNA has been detected in various ophthalmic lesions of the ocular surface and even in phenotypically normal conjunctiva (30,31). HPV infection has been strongly correlated with the pathogenesis and recurrence of conjunctival papillomas, conjunctival intraepithelial neoplasia (CIN) and even squamous cell carcinoma of the conjunctiva (SCCC) (32–36). HPV may also co-exist in SCCC lesions with other oncogenic viruses, such as HIV, thus it is difficult to determine the importance of HPV per se in the development of these lesions (36). The fact that pterygium has also often been reported to co-exist with ocular surface neoplastic lesions (37,38), supports the hypothesis of oncogenic viral infection or co-operation as a pathogenetic model. Obtaining cells from the ocular surface via non-invasive methodologies, including exfoliation cytology techniques (39), may enable the detection of HPV-infected pterygia.

## 6. Methodologies for HPV detection in pterygium

HPVs are non-enveloped viruses with icosahedral symmetry, composed of a circular double-stranded DNA genome. HPVs cause infections of the skin and the mucous membranes of the anogenital region and the oropharynx. Over 100 types have been fully sequenced and some seem to play an important role in the development of tumors. According to their oncogenic potential, HPVs are divided into low- and high-risk types (oncogenic/high-risk types: 16, 18, 31, 33, 35, 39, 45, 51, 52, 56, 58, 59, 68, 73, 82 and potentially oncogenic types: 26, 53, 66) (17,40,41).

Diagnosis of the viral infection is based on the detection of the HPV-DNA. However, the mode of sample collection, the quantity of the HPV-DNA of the isolated sample and the use of various HPV-DNA detection techniques with different sensitivity and specificity, are factors that may significantly affect the detection rates of HPV infections (40).

HPV-DNA can be directly isolated from a biopsy specimen with *in situ* hybridization (ISH), Southern blotting and dot blot hybridization. However, these techniques are laborious, need a large quantity of purified DNA and their sensitivity is limited (40).

In cases where the biopsy specimen is small with a limited quantity of HPV-DNA, nucleic acid amplification assays can be used to increase the sensitivity and specificity of the test. Hybrid Capture II (HC-II) is a non-radioactive signal amplification technique, accurate for mucosal lesions, that distinguishes high-risk from low-risk HPV-types, but is not appropriate for genotyping (40–42).

Due to its high sensitivity, polymerase chain reaction (PCR) is frequently associated with a high rate of false-positive results (43). Southern blot, dot blot, reverse dot blot, digestion with restriction endonucleases or direct sequence analysis performed after DNA amplification can help increase the sensitivity and specificity of the test (41,42). Real-time PCR or quantitative PCR (qPCR) permits rapid detection and quantification of the viral load during the various cycles of the PCR process (real-time) (43). Reverse transcriptase-PCR (RT-PCR) is a qualitative assay that permits the identification of viral gene expression with the use of reverse transcriptase. The combination of the two techniques, quantitative RT-PCR or real-time RT-PCR (qRT-PCR), is considered to be the first choice assay for the detection of viral gene expression as it combines quantitative and qualitative advantages of the two methods (44,45).

## 7. Potential therapeutic interventions in HPV-infected pterygium

Current treatment of pterygium includes surgical excision and occasionally adjunctive therapy. Several surgical techniques have been described: bare sclera closure, sliding conjunctival flaps, use of autologous conjunctival and limbal grafts or amniotic membranes (2,46) (Fig. 2). Due to the possible complications and costs of surgical treatment and the risk of recurrence, often aggressive, various adjunctive therapies have been proposed, including β-irradiation and the use of mitomycin C or 5-fluorouracil. However these methods have been associated with corneoscleral necrosis and melting, limbal stem cell deficiency and variable recurrence rates. B-irradiation has also been associated with cataract formation (2,46).

Interferons are a family of proteins with antiviral, antiproliferative, antiangiogenetic and immunomodulatory properties, produced from the organism in response to various stimuli (47). The recombinant form α-2b (IFN-α-2b) has been used for the treatment of condylomata acuminata, chronic hepatitis B and C, Kaposi sarcoma, malignant melanoma, hairy cell leukemia and follicular lymphoma (47). Unlike mitomycin C and 5-fluorouracil, adverse effects associated with the topical or sub-conjunctival administration of IFN-α-2b are less severe (48–52). IFN-α-2b in the form of eye drops has successfully been used thus far in the management of CIN and conjunctival papilloma (48–51). IFN-α-2b has also been reported to prevent the recurrence of pterygium (52). However, additional investigation is required to fully assess the value of this treatment modality in the treatment of pterygium.

## 8. Conclusion

Despite controversies in the medical literature concerning HPV involvement in pterygium (possibly due to racial susceptibility or methodological differences), most studies agree that HPV is detected in at least a sub-group of pterygia and that HPV infection may affect both pathogenesis and clinical behaviour (including recurrence) of these lesions. Accordingly, it would be interesting to explore the possibility of anti-viral medications or even vaccination, which may represent novel options in the therapy of selected, HPV-infected pterygia.

## Figures and Tables

**Table I:** Prevalence of pterygium-associated viruses in various studies.

Authors/(Refs.)	Date of publication	HPV prevalence	HSV prevalence	CMV prevalence	EBV prevalence	Country of the study	Method of detection
Spandidos, *et al*(28)	1994	-	45%	40%	0%	Greece	PCR
Detorakis, *et al*(18)	2001	24%	22%	-	-	Greece	PCR
		(Type 18)		-	-		
Gallagher, *et al*(19)	2001	50%	-	-	-	UK	PCR
		(Types 6,11,16)					
Piras, *et al*(20)	2003	100%	-	-	-	Italy	PCR- sequencing
		21%				Equador	
		(Types 52, 54, *cand*HPV90, unknown)					
Schellini, *et al*(21)	2006	0%	-	-	-	Brazil	PCR
Sjö, *et al*(34)	2007	4.4%^a^	-	-	-	Denmark	PCR-ISH
		(Type 6)					
Chen, *et al*(29)	2008	-	5%^a^	-	-	Taiwan	PCR-ISH
Takamura, *et al*(23)	2008	4.8%^b^	-	-	-	Japan	PCR-HC II
Rodrigues, *et al*(24)	2008	58.3%				Brazil	PCR
		(Types 1, 2, 16)					
Otlu, *et al*(25)	2009	0%	-	-	10%	Turkey	Real-time PCR
Tsai, *et al*(16)	2009	24%	-	-	-	Taiwan	Nested-PCR
		(Types 16, 18)	-	-	-	Poland	PCR
Piecyk-Sidor, *et al*(26)	2009	27.6%					
		(Types 5, 6, 11, 16, 18, 31, 52, 59)					
Hsiao, *et al*(27)	2010	3%	-	-	-	Taiwan	PCR-ISH
		(Type 18)					

a Virus-DNA negative with ISH;

b HPV-DNA negative with Hybrid Capture II.
